# Anelastic Behaviour of Commercial Die-Cast Magnesium Alloys: Effect of Temperature and Alloy Composition

**DOI:** 10.3390/ma14237220

**Published:** 2021-11-26

**Authors:** Hua Qian Ang

**Affiliations:** School of Engineering, RMIT University, Melbourne, VIC 3000, Australia; huaqian.ang@rmit.edu.au

**Keywords:** magnesium alloys, anelasticity, mechanical properties, elastic modulus, yield strength, die casting

## Abstract

The anelastic deformation, resulting from partial reversal of {10$\bar{1}$2} twinning, is studied at room temperature to 150 °C on several commercial die-cast magnesium alloys for the first time. The magnitude of anelastic strain decreases with increasing temperature. For inter-alloy comparison, AZ91 shows the largest maximum anelastic strain, while AM40 and AM60 show similar maximum anelastic strain. The phenomenon is discussed in terms of solid solution softening and hardening of slip planes and how they influence twinning. T5-aged AE44 consistently shows smaller magnitude of anelasticity compared to as-cast AE44, suggesting that the precipitates formed during ageing may decrease the twin-boundary mobility and further suppress untwinning. Presence of anelasticity poses a challenge to yield strength measurement using the conventional 0.2% offset method, and a more accurate and consistent method of using a higher offset strain or a lower modulus is proposed in this study.

## 1. Introduction

The hexagonal closed-packed (HCP) crystal structure of magnesium (Mg) provides only two independent basal slip systems [1], while the non-basal slips (i.e., prismatic and pyramidal slip) only activate at higher stress levels at room temperature (RT) [2,3,4]. To meet the requirements of von Mises’ criterion, which needs five independent systems for homogeneous plastic deformation [5,6], {10$\bar{1}$2} <10$\bar{1}$1> extension twinning is activated at low stresses and strains [7,8]. These {10$\bar{1}$2} twins are unstable in the loaded condition [9], and they can partially revert upon unloading [10]. The partial reversal of {10$\bar{1}$2} twinning is the main cause for anelasticity in Mg and Mg alloys [11,12,13], manifesting as large hysteresis loops in the cyclic stress–strain curve as observed in pure Mg [11], Mg–Zn alloys [14,15], Mg–Gd alloys [16], Mg–Al alloys [12,17], AZ31 (Mg–3Al–1Zn, all compositions in weight percent hereafter unless specified) [18], AZ91 (Mg–9Al–0.6Zn) [19], AM60 (Mg–6Al–0.3Mn) [20] and AE44 (Mg–4Al–4RE) [21].

Anelasticity allows Mg and Mg alloys to deform reversibly beyond the linear elastic region, and this poses a challenge to conventional yield strength measurement [22]. Moreover, with a linear stress–strain relationship, a single elastic modulus value can easily be determined, but with the presence of anelasticity (non-linear stress–strain relationship), the secant elastic modulus (effective modulus) has been shown to vary with strain [20]. This creates a problem for the engineering designs that are based on a constant value of the elastic modulus. Hence, the study of anelastic deformation is important to understand the stiffness and yielding behaviour of Mg alloys.

High-pressure die-cast alloys constitute for over 90% of Mg alloy usage [23,24], and they exhibit significant anelastic deformation due to their small grain size. Firstly, small-grained alloy contains fine and unstable twins, and they are more likely to revert upon unloading. Secondly, the number of grains favourably oriented for twinning is also larger for small grain size. These two factors were reported to account for the large anelastic strain in small-grained die-cast alloys [14,19].

Anelastic behaviour is also influenced by the solute content. Anelasticity was observed to be the largest in pure Mg, and anelasticity decreased with increasing Zn [14] and Gd [16] solute concentrations. The effect of solute is, however, different for Mg–Al alloys. In Mg–Al alloys, the anelasticity was observed to be almost similar at low Al solute concentrations up to 2 at.%, increasing again at high Al solute concentrations at 9 at.% [15,25]. The difference in behaviour between Mg–Zn, Mg–Gd, and Mg–Al alloys was attributed to the presence of short-range order (SRO) by the different solutes [15,16]. {10$\bar{1}$2} twinning is shuffling dominated [26], and the presence of SRO will make twinning and untwinning more difficult [27]. Both Zn and Gd in solution have a tendency to develop SRO [28] and thus the monotonic decrease of anelasticity with Zn and Gd solute concentrations [15,16]. In contrast, Al forms near-random solid solutions [29] and does not have any hardening effect on twinning [15,30]. The increased anelastic strain observed in Mg–9Al [15] and AZ91 [31] was ascribed to solid solution hardening of slip systems at high Al concentrations, making twinning more necessary as a deformation mechanism.

This study focuses on commercially available die-cast Mg alloys, namely AE44, AM40, AM60, and AZ91. These alloys were selected primarily due to their wide application in automotive parts, for example, AE44 in front engine cradle and powertrain components, AM40 and AM60 in energy-absorbing components, such as seat structures and instrument panels, and AZ91 in steering column brackets [32,33].

The effect of strain rate on anelasticity of these die-cast alloys has recently been investigated by the present author [30]. Anelasticity was observed to increase with increasing applied strain rates from 10^−6^ to 10^−1^ s^−1^. This was attributed to the delay onset of prismatic slip at higher strain rate, increasing the alloys’ tendency to twin. The anelasticity of AE alloy was also observed to be more strain-rate sensitive than the AM and AZ alloys.

This paper not only serves as an extension to the strain-rate study [30,34], but to the author’s knowledge, this is the first study to report on the effect of elevated temperature on the anelastic deformation for Mg alloys. Although the scope of this work is limited to the temperature range from RT to 150 °C due to limitations of equipment, this study on anelasticity offers an overview of how twinning and untwinning develop during deformation at different temperatures, and how they can be affected by alloy composition. The implications of the temperature dependence of anelasticity and secant elastic modulus on yield strength measurement are also discussed, which may provide a new perspective in future elevated temperature applications of die-cast Mg alloys.

## 2. Materials and Methods

Mg alloys, AE44, AM40, AM60, and AZ91, used in this study were high-pressure die-cast in a cold chamber machine at CSIRO, Melbourne, Australia. Details of the casting parameters were previously reported [35]. Some as-cast AE44 specimens were subject to an ageing treatment at 200 °C for 32 h (labelled T5-aged) in order to improve the strength–ductility combinations due to the precipitation of nanoscale Al–Mn particles upon ageing [36]. Table 1 lists the chemical compositions of the studied alloys analysed using inductively coupled plasma atomic emission spectroscopy (ICP-AES).

**Figure 1:**
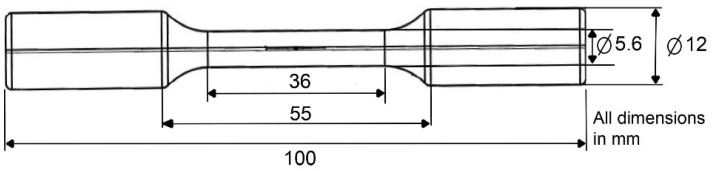
Schematic diagram of the tensile specimen used in this study.

All specimens used in this study are dog-bone shaped with a cylindrical cross-section diameter of 5.6 mm and a 36 mm parallel section in the gauge length, as shown in Figure 1. Monotonic and cyclic tension loading–unloading tests were carried out in a temperature-controlled environmental chamber mounted on an Instron universal testing machine. All tests were carried out at a crosshead speed of 2 mm/min ($\overset{\cdot }{\varepsilon }$ ≈ 10^−3^ s^−1^) at RT of about 22 °C (295 K) and at elevated temperatures of 50 °C (323 K), 100 °C (373 K), and 150 °C (423 K). Temperature was allowed to equilibrate for 5–10 min before testing. To accurately measure the temperature, a K-type thermocouple was bent and inserted between the grips of the machine, so that it was contacting the specimen. Temperature variations of 1–2 °C were observed during testing.

The cyclic tension loading–unloading tests were strain-controlled, loaded to a predetermined strain, unloaded to zero stress, and then reloaded again following the International Standards Organization (ISO) standard [37]. All alloys were cyclic tested to 3% strain, except AZ91, which was tested to a larger strain of 5% to determine the saturation of anelastic strain. An extensometer was attached to the specimen gauge area to record the strain values during testing. Each test was repeated two to three times, and good reproducibility of data was observed. Compression testing is not included in this study, as high-pressure die-cast alloys are known to be isotropic in behaviour [38,39].

Microstructures were characterised by a scanning electron microscopy (SEM) equipped with an energy dispersive X-ray (EDX) spectroscopy and an electron backscatter diffraction (EBSD). EDX line scan was conducted to measure the Al solute concentration level in the α-Mg matrix. EBSD data were collected at 20 kV with a 0.5 μm step size to reveal the deformation twins. For each sample, three locations were analysed. Samples for microstructural analysis were polished with 600 and 2400 SiC papers followed by 3 and 1 μm alcohol-based diamond suspensions and were finished by a 0.06 μm colloidal silica suspension.

## 3. Results

**Figure 2:**
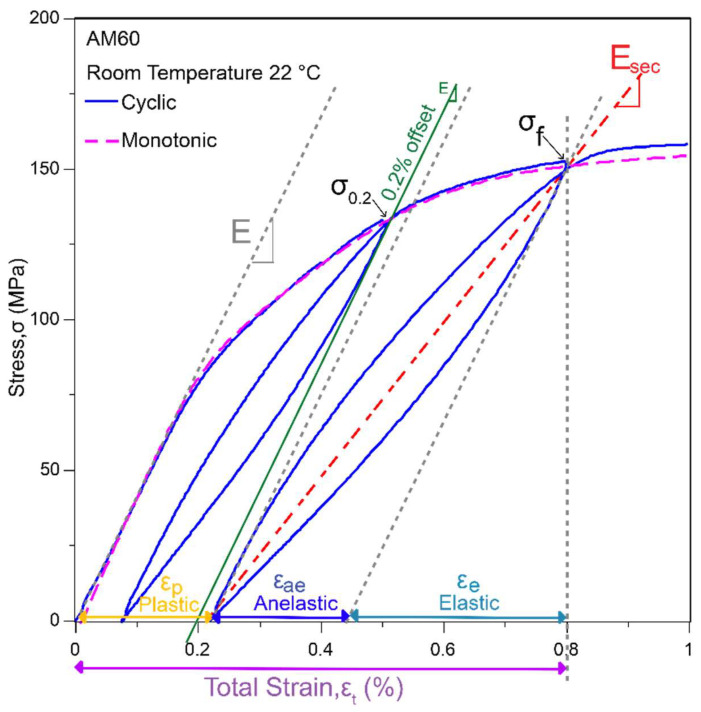
Cyclic tension loading-unloading test of die-cast AM60 at RT. The total strain (ε_t_) is made up of linear elastic strain (ε_e_), anelastic strain (ε_ae_), and plastic strain (ε_p_). E is the elastic modulus, while E_sec_ is the secant elastic modulus. σ_f_ is the applied stress at the start of unloading, and σ_0.2_ is the 0.2% offset yield stress.

A typical cyclic loading–unloading tensile curve is shown in Figure 2, whereby several parameters used in this study are defined. The non-linearity in the unloading curve forms a closed loop upon reloading. If the specimen is unloaded after some applied plastic strain, a larger unloading strain is required to achieve the same amount of plastic strain as under linear elastic behaviour. The monotonic tensile curve also matches with the cyclic curve, indicating that cyclic loading does not have any significant effect on the work-hardening behaviour. The 0.2% offset line illustrates the yield strength measurement method for Mg and Mg alloys based on the American Society for Testing and Materials (ASTM) [40] and ISO [37] standards.

**Figure 3:**
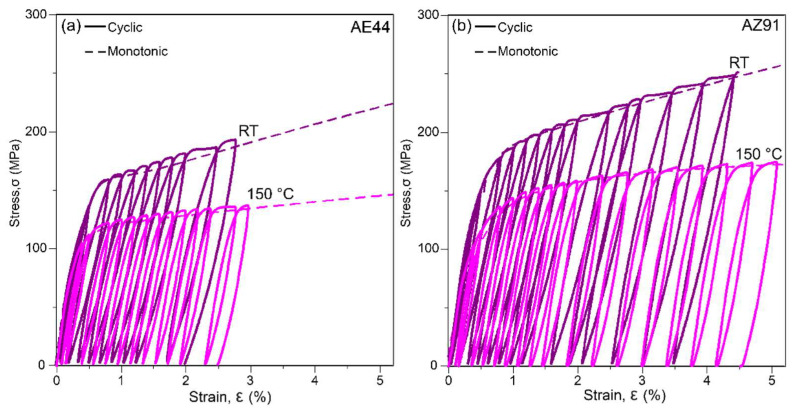
Examples of hysteresis loops in as-cast (**a**) AE44 and (**b**) AZ91 cycled in tension at RT and 150 °C. Dashed line represents the corresponding monotonic flow curve.

Similar cycles for as-cast AE44 and AZ91 at RT and 150 °C are shown in Figure 3. As expected, the flow stress decreases with increasing temperature as the alloy becomes more ductile at higher temperature (tensile properties are presented in Table 2). The hysteresis loops also become smaller as temperature increases. Similar behaviour was observed in T5-aged AE44, AM40 and AM60 (not shown). Some AZ91 specimens tested at RT fractured before reaching 5% strain due to the inherent low RT tensile ductility of the alloy.

**Figure 4:**
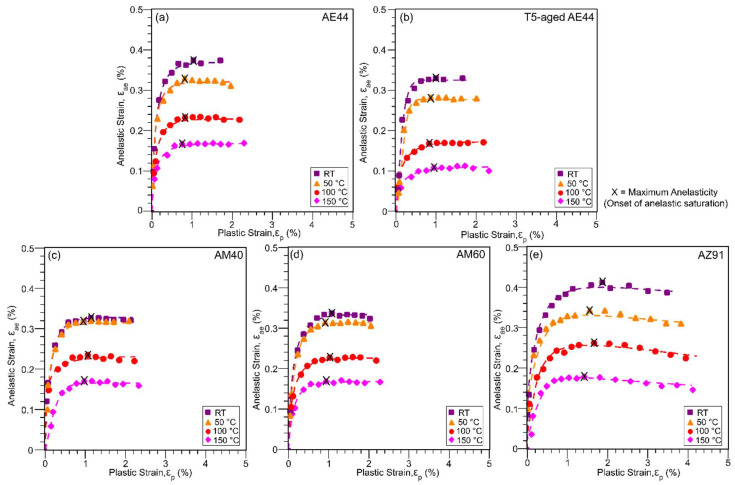
Anelastic strain, ε_ae_, as a function of the applied plastic strain, ε_p_, for (**a**) AE44, (**b**) T5-aged AE44, (**c**) AM40, (**d**) AM60, and (**e**) AZ91, at RT and elevated temperatures. Maximum anelasticity indicates the onset of anelastic saturation, as marked by the symbol ‘X’.

**Figure 5:**
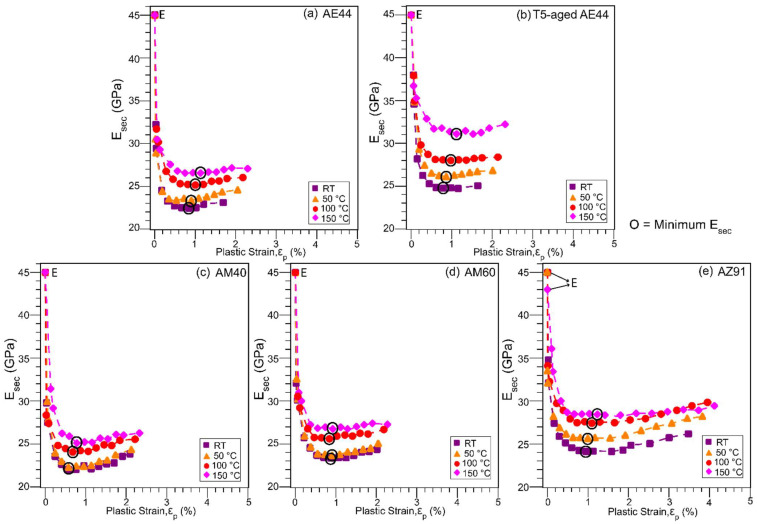
The secant elastic modulus, E_sec_, as a function of the applied plastic strain, ε_p_, for the alloys and temperatures of Figure 4. E is the elastic modulus at zero plastic strain, estimated from a low stress region of 30 MPa. The symbol ‘O’ denotes the minimum E_sec_.

The anelastic strain (ε_ae_, defined in Figure 2) measured from the width of the hysteresis loop and the secant elastic modulus (E_sec_, in Figure 2) are plotted as a function of the applied plastic strain for the studied alloys and temperatures in Figure 4 and Figure 5, respectively. In Figure 4, the anelastic strain develops gradually after a small applied plastic strain then reaches a maximum and saturates at between 0.1% (T5-aged AE44, 150 °C) and 0.4% (AZ91, RT), depending on the alloy and temperature. The maximum anelasticity also denotes the onset of anelastic saturation, and it is marked by the symbol ‘X’. In AE and AM alloys, the anelasticity saturates after a plastic strain of about 0.8–1%, whilst the AZ91 alloy saturates at a larger plastic strain of about 1.4% (at 150 °C)–1.8% (at RT), and hence AZ91 was cyclic tested to a larger strain to see this plateau effect. A slight decrease in anelasticity is also observed in AZ91 beyond 2.4% plastic strain; strains applied at other alloys are not sufficient to show this effect. In Mg–Al–RE alloy, T5-aged AE44 exhibits a smaller maximum anelasticity as compared to as-cast AE44, whereas in the Mg–Al alloys, AM40 and AM60 exhibit similar maximum anelasticity, whilst AZ91 has the largest maximum anelasticity at a given temperature. Overall, the magnitude of anelasticity decreases with increasing temperature for all the alloys.

In Figure 5, all the alloys start off with an elastic modulus (E, in Figure 2) of 45 GPa regardless of the temperatures, except AZ91 at 150 °C which has a slightly smaller E-value of about 43 GPa. Although the elastic modulus of Mg is normally taken as 45 GPa [41] as the nominal value, the elastic modulus in Mg alloys can vary, depending on the metallurgical conditions, but it remains almost constant in the range of 39–45 GPa at RT [42] and up to 200 °C [43]. The modulus then decreases significantly with temperature [44], i.e., dropping to about 27 GPa at 225 °C in AZ31 [45]. Note that determining the elastic modulus in Mg alloys is difficult due to the small linear elastic region (<40 MPa). As reported by [22], the elastic modulus decreases when a higher stress level is used in modulus determination. For consistency, the E-value in this study is estimated from a stress level of 30 MPa. The secant elastic modulus (modulus value determined from the subsequent loops) then decreases with increasing applied plastic strain, reaching a minimum at about 0.6–1.2% plastic strain (as marked by symbol ‘O’), slightly increasing afterwards. For all the alloys, the modulus consistently drops to a smaller value at RT, a consequence of loops being larger at RT (Figure 3). Both T5-aged AE44 and AZ91 are high-strength alloys and they exhibit a smaller decrease in modulus in general, in agreement with previous observations for high-strength alloys [14,19].

**Figure 6:**
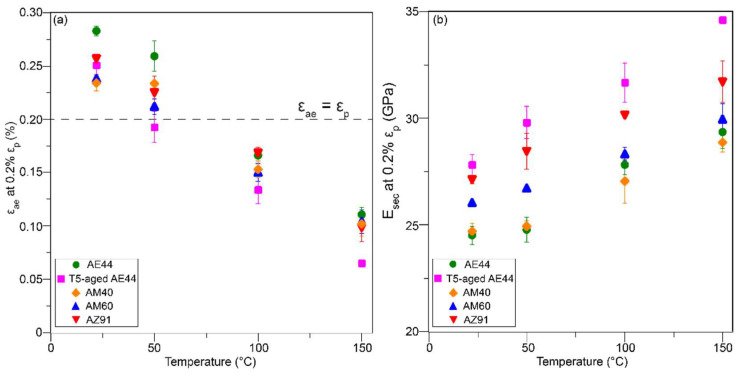
The magnitude of (**a**) anelastic strain, ε_ae_, and (**b**) secant elastic modulus, E_sec_, at 0.2% plastic strain, ε_p_. The values shown are averaged from two to three repeated tests. Dashed line shown in (**a**) indicates ε_ae_ = ε_p_.

Yielding is often defined by the stress at which 0.2% plastic strain occurs. Therefore, it is important to quantify the amount of anelastic strain and secant elastic modulus at 0.2% plastic strain, as shown in Figure 6a,b, respectively. Note that it is impossible to pre-identify the levels of stress and strain at which 0.2% plastic deformation occurs. In many cases, the hysteresis loops unloaded close to, but not exactly, 0.2% plastic strain during testing. In such cases, the anelastic strain and secant elastic modulus in Figure 6 are interpolated between the adjacent values (values measured from loops unloading to plastic strains before and after 0.2%), leading to some deviations in the data. It is interesting to observe some amount of anelasticity at such a small plastic strain of 0.2%; in particular, the anelastic component can be much larger than the plastic component at RT and 50 °C (above the dashed line in Figure 6a). The secant elastic modulus at 0.2% plastic strain is also much smaller than the nominal elastic modulus of Mg and Mg alloys, which is 45 GPa [41].

**Figure 7:**
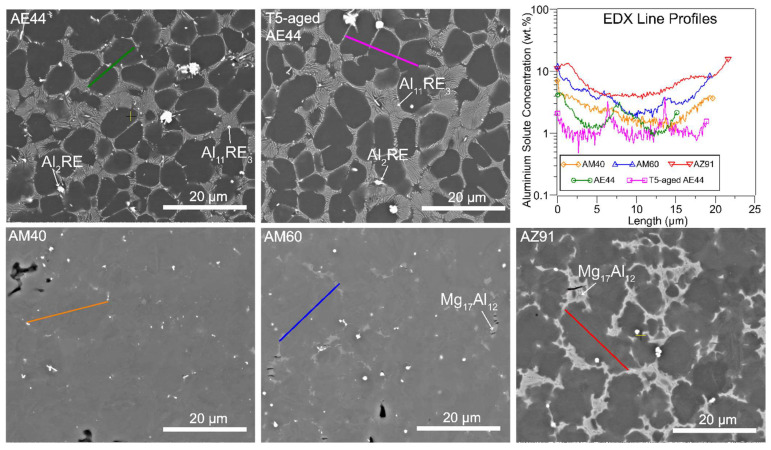
Backscattered SEM micrographs and EDX line profiles of the α-magnesium matrix.

The typical microstructures of the studied alloys are shown in Figure 7, which are characterised by primary α-Mg dendrites (dark) surrounded by the eutectic consisting of intermetallic phase (light) in the interdendritic regions. For AE44 and T5-aged AE44, the dominant intermetallic phase is Al_11_RE_3_, which has a lamellar morphology and some minor Al_2_RE phase which has a polygonal shape. It is interesting to see no changes to the intermetallic phases after T5 ageing. For AM40, AM60, and AZ91, the intermetallic phase is Mg_17_Al_12_ [46,47], which appears as discrete particles surrounded by supersaturated eutectic α-Mg. Detailed characterisation of these types of intermetallic phases can be found in previous work [48,49,50].

The volume fraction of the brittle Mg_17_Al_12_ phase increases with increasing aluminium alloying, leading to an increase in hardness but reduction in ductility in AZ91. The distribution of Al solute in the α-Mg matrix across the regions indicated in the SEM micrographs was measured by EDX and plotted in Figure 7. There is an increase in the Al solute concentration from the centre of the dendrite cells towards the boundaries. This is expected, as the eutectic containing intermetallic phases solidifies last during casting, and so it is richer in Al than the primary α-Mg dendrites. The Al solute concentration near the dendrite boundaries and at the centre of the dendrite cells is recorded in Table 3.

**Figure 8:**
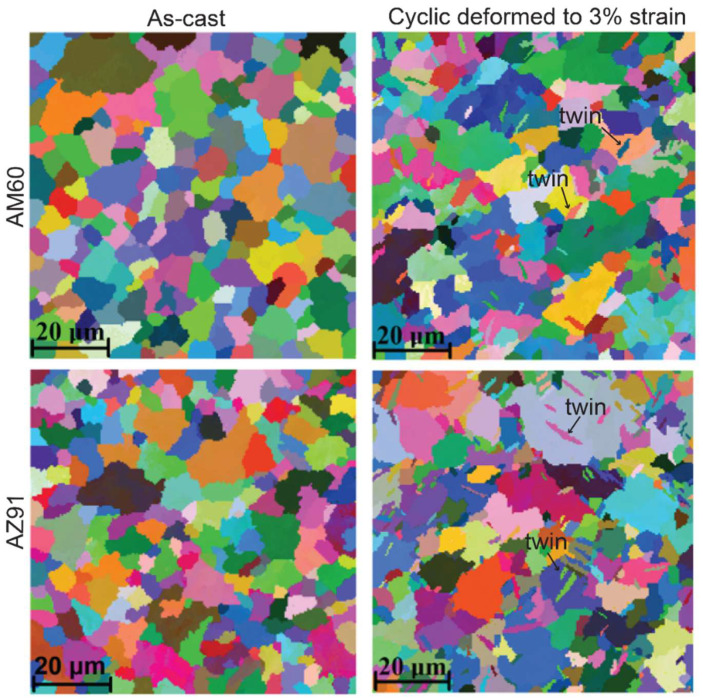
EBSD maps of AM60 and AZ91 showing twin-free as-cast microstructure and twin formation in deformed microstructure.

EBSD maps of the as-cast and deformed microstructures after cyclic testing to 3% strain at RT for AM60 and AZ91 are shown in Figure 8. It is clear that the as-cast microstructure is twin-free, and twins formed after cyclic deformation. The volume fraction of twinning in AZ91 is higher than AM60 and other alloys (not shown). Note that the twins shown in Figure 8 are the reverted twins because the samples were unloaded before EBSD analysis. These twins can partially revert by becoming larger upon reloading, initiating the anelastic property in Mg alloys. All the studied alloys have similar grain sizes (≈8 μm) to eliminate grain size effect.

## 4. Discussion

The anelasticity behaviour observed in Figure 4, which increases with plastic strain and becomes saturated at a larger plastic strain, is consistent with previously published work [19,51]. When the deformation is small, below a plastic strain of 0.8–1% in AE and AM alloys or 1.4–1.8% in AZ91, twins can multiply undisturbedly upon formation, and anelasticity increases with applied strain. When deformation is large, an increase in slip dislocation and twinning activity in the surrounding matrix can reduce the twin-boundary mobility, making untwinning more difficult [52], and consequently, saturating and eventually decreasing the anelastic strain (as observed in AZ91 beyond 2.4% plastic strain) as deformation continues. Note that the overall behaviour of E_sec_ (Figure 5) corresponds to that of anelasticity (Figure 4), whereby an increase in anelasticity leads to a decrease in E_sec_ as the loops become larger, and vice versa. The minimum in E_sec_ and the maximum in anelastic strain occur at a slightly different plastic strains due to the work-hardening effect. The following discussion will now consider how temperature and alloy composition influence the anelastic behaviour and consequently affecting the yield strength measurement.

### 4.1. The Effect of Temperature

Present results show that anelasticity consistently decreases with increasing temperature (Figure 4); this applies even at very low plastic strain of 0.2% (Figure 6a), for all the studied alloys. This can be rationalised as follows. At RT deformation, Mg and Mg alloys have a limited number of basal slip systems [1]. Other non-basal slip systems, such as prismatic slip, first order and second order pyramidal slip are also less favoured as their critical resolved shear stresses (CRSSs) at RT are several orders of magnitude greater than that of basal slip and twinning [53,54]. Therefore, to meet the von Mises’ criterion, which requires five independent systems for homogeneous deformation [6], {10$\bar{1}$2} twinning, which has the smallest CRSS, is profusely activated, magnifying the anelasticity at RT.

As temperature increases, the CRSSs of non-basal slip systems are known to decrease significantly even to a smaller level than the CRSS of {10$\bar{1}$2} twinning [53,55]. This is because, unlike the non-basal slip which is highly sensitive to temperature [56,57], the CRSS of {10$\bar{1}$2} twinning is not temperature sensitive [58,59]. Die-cast alloys have random texture, and no specific deformation mechanism is favoured [60,61,62]; therefore, each deformation mechanism, such as slip and twinning, will be activated when the local stress acting upon them reaches their CRSS. As the non-basal slip systems become easier to activate at higher temperature, while {10$\bar{1}$2} twinning is not affected by temperature, the relative propensity for twinning and untwinning decreases, thereby reducing the maximum anelasticity with increasing temperature. At low plastic strain of 0.2% (Figure 6a), the larger amount of anelasticity compared to plasticity at RT–50 °C suggests that twinning is the dominant deformation mode at least up to 50 °C; non-basal slip may become increasingly prominent beyond 50 °C as indicated by the smaller anelasticity than plasticity at 100–150 °C. In fact, it has been widely reported that the improved ductility of Mg alloys at elevated temperature (Table 2) is the result of the activation of these non-basal slip systems [63,64].

### 4.2. The Effect of Alloy Composition

In Mg–Al alloys without RE addition AM40, AM60 and AZ91, AZ91 exhibits the largest maximum anelasticity, while AM40 and AM60 appear to have similar maximum anelasticity at a given temperature (Figure 4). Since the studied alloys have similar grain sizes, the main difference is the Al concentration. AZ91 has the highest Al solute concentration in the α-Mg matrix compared to AM60 and AM40 as shown in Figure 7. Firstly, high Al concentration produces solid solution hardening of slip planes (increase the CRSS of slip), but Al has little hardening effect on twinning [15,30], the net result being more twinning is activated to assist plastic deformation. This correlates well with the EBSD analysis in Figure 8, which shows higher volume fraction of twinning in AZ91 than AM60. Secondly, increasing Al concentration increases the twin growth stress (suppresses twin growth), resulting in formation of numerous smaller and unstable twins [17]. These two effects can increase the tendency of untwinning, magnifying the maximum anelasticity in AZ91. Since slip becomes more difficult to activate in AZ91, twins can easily untwin without the interference of slip (abundance of slip dislocation can decrease twin-boundary mobility and untwinning [52]) until slip is profusely activated at higher stresses and strains. This explains the delayed saturation of anelasticity at ~1.4–1.8% plastic strain in AZ91 as compared to ~0.8–1% in other alloys. Note that ease of activation of non-basal slip at 150 °C in AZ91 may counteract the hardening effect of Al, resulting in saturation of anelasticity at a smaller plastic strain of ~1.4% compared to 1.8% at RT.

The similar anelasticity between AM40 and AM60 can be explained as follows. A small addition of Al solute up to 0.5 wt.% was confirmed to soften the prismatic plane (decrease the CRSS of prismatic slip); higher solute level was not investigated in [65]. However, later work by Nagarajan et al. [15] showed softening of prismatic plane up to 2 wt.% of Al; hence, the present work suggests that prismatic plane may undergo a transition from softening to hardening with increasing Al concentration from AM40 to AZ91. It is likely that AM40 and AM60 are in the softening–hardening transition region in which Al has little effect on prismatic slip. Since Al forms near-random solid solutions [29] and also does not have any hardening effect on twinning [15,30], and hence, the similar maximum anelasticity of AM40 and AM60 at all temperatures.

In Mg–Al–RE alloy AE44, T5 ageing may also influence the anelastic deformation, as T5-aged AE44 consistently shows smaller maximum anelasticity, regardless of the temperatures. Although AE44 and T5-aged AE44 have similar intermetallic phases, there was a formation of Al–Mn nanoscale precipitates after T5 ageing, as reported recently [66]. These precipitates may serve as obstacles to twinning and untwinning as twins propagate along the twin boundaries, decreasing the twin-boundary mobility. Hence, untwinning becomes more difficult and decreases the maximum anelasticity in T5-aged AE44.

Note that it is more complex to compare between Mg–Al–RE and Mg–Al alloys due to microstructural difference, as shown in Figure 7, and it may not provide meaningful results. Besides, studies showed that the degree of interconnection of the percolating intermetallic network of Mg_17_Al_12_ has a measurable effect on the work hardening [67,68] and the anelastic deformation [69]. It is, however, not known if the Al–RE intermetallic network has the same effect.

### 4.3. The Effect of Anelasticity and E_sec_ on Yield Strength Measurement

**Figure 9:**
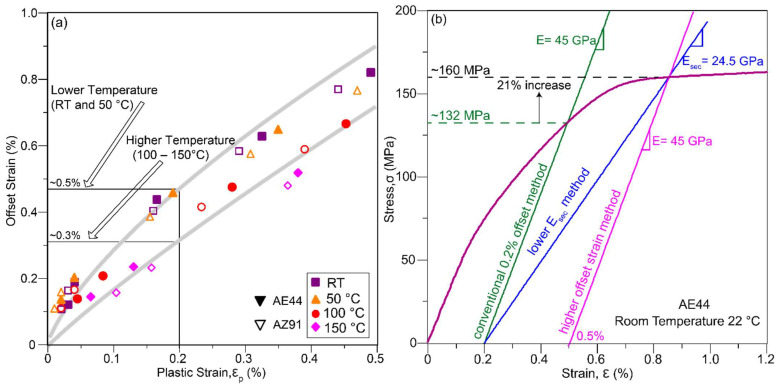
(**a**) Appropriate offset strains (plastic strain + anelastic strain) for as-cast AE44 and AZ91 at room and elevated temperatures, and (**b**) comparison of proposed yield strength measurement methods (higher offset strain and lower E_sec_ methods) with the conventional 0.2% offset method.

The yield strength of materials is generally measured by offsetting the linear elastic modulus, E to some amount of permanent plastic strain. The amount of permanent plastic strain can range from 0.1% for ferrous to 0.5% for non-ferrous materials [37], and 0.2% is used for Mg and Mg alloys. However, unloading at 0.2% offset yield stress, σ_0.2_, leaves only a very small fraction of permanent plastic strain (<0.1%) due to the reversible anelastic component of Mg and Mg alloys, as illustrated in Figure 2. Clearly, this conventional 0.2% offset method underestimates the yield strength of Mg alloys without considering the anelastic effect. To accurately measure the 0.2% permanent strain yield stress, elastic modulus of Mg (normally taken as 45 GPa) should be offset to a higher strain value. This higher offset strain method should consider the anelasticity at 0.2% plastic strain [22]. Figure 9a is a conversion chart which shows the appropriate offset strains (plastic strain plus anelastic strain) for as-cast AE44 (solid symbol) and AZ91 (hollow symbol). The offset strain to achieve a 0.2% permanent strain upon unloading can range from 0.3% for alloys deforming at higher temperatures (100–150 °C) to 0.5% for alloys deforming at low temperatures (RT–50 °C). This range is also applicable for other studied alloys (not shown), as the anelasticity at 0.2% plasticity (Figure 6a) is quite similar between the alloys, but different between the temperatures.

Alternatively, a lower E_sec_ (Figure 6b) should be applied at 0.2% strain to achieve a more diagnostic property value than the widely used elastic modulus of 45 GPa. Both the proposed higher offset strain and lower E_sec_ methods are compared with the conventional 0.2% offset method in Figure 9b, and their measured yield stress values are reported in Table 2. Higher offset strain method crosses the flat part of the curve, and it provides a more consistent yield stress value as noted by the smaller sample to sample variations (Table 2) than the 0.2% offset method. From Table 2 and Figure 9b, it is clear that the yield strength of the studied alloys is largely underestimated by up to 20% using the 0.2% offset method. The 0.2% offset method may be applicable to most steel and aluminium which have only linear elastic and plastic properties, but in Mg and Mg alloys, higher offset strain or lower E_sec_ methods should be employed to achieve a more accurate yield stress value which leaves a permanent plastic strain close to 0.2% upon unloading.

## 5. Conclusions

The anelastic deformation, attributed to partial reversal of {10$\bar{1}$2} twinning, of several commercial die-cast Mg alloys has been studied from room temperature to 150 °C under cyclic tension loading–unloading testing. The following conclusions can be drawn.

1. The magnitude of anelastic strain decreases with increasing temperature. This is ascribed to the ease of activation of non-basal slip at temperatures beyond 50 °C, reducing the propensity for twinning to occur as a deformation mechanism, and thereby lowering the amount of reversible twinning. The improved tensile ductility of the alloys at elevated temperature is the result of the activation of non-basal slip systems.

2. In Mg–Al alloys, AZ91 consistently shows the largest maximum anelasticity, while AM40 and AM60 have similar anelasticity at all temperatures. The effects can be understood in terms of solid solution hardening and softening of slip system by the Al solute, leading to a change in twinning and untwinning (anelasticity) activity.

3. In Mg–Al–RE alloy, T5-aged AE44 consistently exhibits smaller maximum anelasticity compared to as-cast AE44. T5 ageing forms Al–Mn nanoscale precipitates, and these precipitates may decrease twin-boundary mobility, making partial reversal of twinning more difficult.

4. Anelasticity observed in Mg alloys leads to inaccuracy in yield strength measurement using the conventional 0.2% offset method. Higher offset strain or lower E_sec_ methods, which account for the anelastic effect, are proposed to improve the accuracy of the yield strength measurement for die-cast magnesium alloys.

## Figures and Tables

**Table 1:** Chemical compositions of the studied Mg alloys in wt.%.

Alloy	Al	Mn	RE (Ce + La)	Zn	Mg
AE44	3.67	0.31	3.83	<0.01	Bal.
AM40	4.44	0.21	<0.01	0.05	Bal.
AM60	6.26	0.29	<0.01	0.1	Bal.
AZ91	8.88	0.19	<0.01	0.74	Bal.

**Table 2:** Monotonic tensile properties of studied die-cast Mg alloys at room and elevated temperatures.

Alloy	Temperature (°C)	Yield Strength by 0.2% Offset Method (MPa)	Yield Strength by Higher Offset Strain Method ^a^ (MPa)	Tensile Strength (MPa)	Elongation to Fracture (%)
AE44	RT	133.0 ± 1.4	161.5 ± 2.1	292.5 ± 6.3	12.4 ± 0.8
	50	126.0 ± 5.3	152.7 ± 2.5	294.0 ± 5.6	20.0 ± 2.9
	100	116.0 ± 2.8	125.5 ± 2.1	246.0 ± 2.8	29.3 ± 1.9
	150	106.0 ± 1.4	111.5 ± 0.7	194.5 ± 0.7	38.1 ± 1.6
T5-aged AE44	RT	167.0 ± 4.2	194.5 ± 0.7	314.0 ± 8.4	11.4 ± 1.9
	50	146.5 ± 2.1	181.0 ± 0.3	300.0 ± 0.0	15.5 ± 0.2
	100	144.5 ± 2.1	156.5 ± 2.1	254.0 ± 5.6	30.8 ± 2.3
	150	127.0 ± 2.8	134.0 ± 2.8	194.5 ± 2.1	30.7 ± 1.8
AM40	RT	111.8 ± 1.7	132.5 ± 3.0	287.0 ± 8.8	17.8 ± 1.8
	50	109.5 ± 3.5	131.0 ± 1.4	281.0 ± 1.4	17.3 ± 0.4
	100	99.3 ± 1.2	109.0 ± 1.7	241.7 ± 7.2	20.9 ± 2.6
	150	80.0 ± 0.0	89.0 ± 1.4	177.0 ± 1.4	27.6 ± 1.3
AM60	RT	126.0 ± 1.4	148.0 ± 0.2	291.5 ± 9.1	13.7 ± 1.4
	50	124.5 ± 0.7	149.0 ± 0.1	300.0 ± 2.8	16.1 ± 0.4
	100	113.0 ± 1.4	124.5 ± 3.5	278.0 ± 5.6	20.7 ± 2.3
	150	94.5 ± 2.1	105.5 ± 0.7	206.0 ± 1.4	25.2 ± 1.2
AZ91	RT	163.0 ± 2.8	185.5 ± 0.7	274.6 ± 13.3	6.6 ± 1.3
	50	140.5 ± 0.7	173.5 ± 0.7	258.5 ± 0.7	6.3 ± 0.5
	100	124.5 ± 0.7	139.5 ± 0.7	270.0 ± 11.3	11.1 ± 1.6
	150	111.0 ± 6.0	125.0 ± 4.3	220.3 ± 13.1	20.0 ± 1.8

^a^ Higher offset strains of 0.5% and 0.3% are applied for lower-temperature deformation RT—50 °C and elevated-temperature deformation 100–150 °C, respectively.

**Table 3:** Al solute concentration level (wt.%) in the α-Mg matrix obtained by EDX.

Alloy	Near Boundaries	Centre
AE44	2–4	1.7
T5-aged AE44	2–3	1.3
AM40	4–7	3.2
AM60	8–11	4.2
AZ91	7–15	7.4

## Data Availability

The raw/processed data required to reproduce these findings cannot be shared at this time, as the data also form part of an ongoing study.
